# Lovastatin Treatment of a Patient with a *De Novo SYNGAP1* Protein Truncating Variant

**DOI:** 10.1089/cap.2018.0159

**Published:** 2019-05-07

**Authors:** Edwin H. Cook, Jayson T. Masaki, Stephen J. Guter, Fedra Najjar

**Affiliations:** ^1^ Department of Psychiatry, Institute for Juvenile Research, University of Illinois at Chicago, Chicago, Illinois.

**To the Editor:**

This report describes the response of a patient with autism spectrum disorder (ASD) and moderate intellectual disability (ID) with a *de novo* protein truncating variant (PTV) in *SYNGAP1* to lovastatin. The patient is a 32-year-old European American man with ASD, confirmed by ADI-R (Le Couteur et al. 1989), ADOS (Lord et al. 1989), and clinical judgment with moderate ID (ratio nonverbal IQ at age 8 years 9 months of 36), and irritability related to an anxious obsessive insistence on sameness and sensory hypersensitivity. He does not have a history of seizures. He has been treated by the same child and adolescent psychiatrist (EHC) for >13 years, with risperidone initiated in 2005 due to irritability. He was stably treated with ziprasidone 80 mg twice daily, and escitalopram 10 mg twice daily at stable doses after August 2013, except for failed cross-titrations from ziprasidone to aripiprazole (August 2017) or risperidone (November 2017) as well as gradual increases in clonidine from 0.1 to 0.3 mg to treat insomnia.

As a subject in The Autism Simplex Collection (Buxbaum et al. 2014), a *de novo* PTV in *SYNGAP1* was identified by the Autism Sequencing Consortium (hg19 chr6:33408610:TC>T, frameshift) (De Rubeis et al. 2014). *SYNGAP1* PTVs are a known etiology of ASD and ID. Preclinical study of other *SYNGAP*1 PTVs demonstrated an impairment in the role of SynGAP in long-term potentiation (LTP) with SynGAP knockdown leading to increased basal Ras activity and impairment of SYNGAP-Ras signaling (Araki et al. 2015). Recent studies have shown that lovastatin regulates Ras signaling and ameliorates synaptic spine pathophysiology, including abnormal spine enlargement and LTP occlusion, resulting from SynGAP knockdown in rat hippocampal primary cultures (Yoichi Araki and Richard Huganir, pers. comm.).

Based on the preclinical findings described earlier, lovastatin, 10 mg daily, was added with the previous medication regimen held constant beginning in July 2016. After 1 month the dose was increased to 20 mg. After 12 weeks, his parent-rated Aberrant Behavior Checklist-Community (ABC-C) total score, Repetitive Behaviors Scale-Revised (RBS-R) total score, and his Social Reciprocity Scale, revision 2 (SRS-2) total *t*-score had improved (Table 1). Lovastatin was then increased to 40 mg daily. However, his ABC-C total score worsened relative to 20 mg daily, with parent report of increased irritability leading to lovastatin discontinuation after 1 month on 40 mg daily.

**Table 1.** Response to Lovastatin

*Months from baseline*	*Lovastatin (mg/day)*	*ABC-C total*	*RBS-R total*	*SRS-2 total* t*-score*
0	0	34	30	81
3	20	26	22	69
4	40	35	24	66
19^a^	0	30	28	71
21	20	25	13	62
26	20	10	18	63
28	20	9	10	58

^a^ Lovastatin was stopped after 4-month rating on 40 mg lovastatin and restarted at 20 mg after the 19-month rating.

ABC-C, Aberrant Behavior Checklist-Community; RBS-R, Repetitive Behaviors Scale-Revised; SRS-2, Social Reciprocity Scale, Revision 2.

Because of the possibility that 40 mg daily was too high, lovastatin was reinitiated in February 2018 at 20 mg daily. His ratings improved again on 20 mg daily (Table 1). His Peabody Picture Vocabulary Test, Fourth Edition scores did not change in response to lovastatin. His parents reported an increased interest in his environment corresponding with rating improvement. As one example, his usual fixation on seeing lawnmowers at a familiar store was replaced with curious exploration of the rest of the store. Notably, this increased range of interests occurred at the same time as improvements in hyperactivity and concentration without loss of pleasure in his preferred activities.

Further titration will be necessary to determine the optimal dose of lovastatin for this individual patient. It is also notable that he continues to have anxiety related to sensory hypersensitivity, although he may be better able to communicate this to his parents to prevent episodes of aggression. It is not yet clear that his ziprasidone may be reduced after his improvement on lovastatin.

Statins are being investigated in the treatment of neurofibromatosis (Stivaros et al. 2018) and Fragile X syndrome. The potential for treatment effects in an adult patient with *SYNGAP1*-associated ASD and ID are supported by evidence that SynGAP functions throughout development and restoration of function in adult mouse brain improves cognitive function (Creson et al. 2018). However, an obvious limitation of a single case report is the possibility of expectancy effect and a randomized placebo-controlled trial will be necessary to confirm this singe case observation.

## References

[B1] Araki Y, Zeng M, Zhang M, Huganir RL: Rapid dispersion of SynGAP from synaptic spines triggers AMPA receptor insertion and spine enlargement during LTP. Neuron 85:173–189, 2015 25569349 10.1016/j.neuron.2014.12.023 PMC4428669

[B2] Buxbaum JD, Bolshakova N, Brownfeld JM, Anney RJ, Bender P, Bernier R, Cook EH, Coon H, Cuccaro M, Freitag CM, Hallmayer J, Geschwind D, Klauck SM, Nurnberger JI, Oliveira G, Pinto D, Poustka F, Scherer SW, Shih A, Sutcliffe JS, Szatmari P, Vicente AM, Vieland V, Gallagher L: The Autism Simplex Collection: An international, expertly phenotyped autism sample for genetic and phenotypic analyses. Mol Autism 5:34, 2014 25392729 10.1186/2040-2392-5-34 PMC4228819

[B3] Creson T, Rojas C, Hwaun E, Vaissiere T, Kilinc M, Holder JL, Tang J, Colgin LL, Miller CA, Rumbaugh G: Re-expression of SynGAP protein in adulthood improves translatable measures of brain function and behavior in a model of neurodevelopmental disorders. bioRxiv 474965, 2018 10.7554/eLife.46752 PMC6504227 31025938

[B4] De Rubeis S, He X, Goldberg AP, Poultney CS, Samocha K, Cicek AE, Kou Y, Liu L, Fromer M, Walker S, Singh T, Klei L, Kosmicki J, Shih-Chen F, Aleksic B, Biscaldi M, Bolton PF, Brownfeld JM, Cai J, Campbell NG, Carracedo A, Chahrour MH, Chiocchetti AG, Coon H, Crawford EL, Curran SR, Dawson G, Duketis E, Fernandez BA, Gallagher L, Geller E, Guter SJ, Hill RS, Ionita-Laza J, Jimenz Gonzalez P, Kilpinen H, Klauck SM, Kolevzon A, Lee I, Lei I, Lei J, Lehtimaki T, Lin CF, Ma'ayan A, Marshall CR, McInnes AL, Neale B, Owen MJ, Ozaki N, Parellada M, Parr JR, Purcell S, Puura K, Rajagopalan D, Rehnstrom K, Reichenberg A, Sabo A, Sachse M, Sanders SJ, Schafer C, Schulte-Ruther M, Skuse D, Stevens C, Szatmari P, Tammimies K, Valladares O, Voran A, Li-San W, Weiss LA, Willsey AJ, Yu TW, Yuen RK, Study DDD, Homozygosity Mapping Collaborative for A, Consortium UK, Cook EH, Freitag CM, Gill M, Hultman CM, Lehner T, Palotie A, Schellenberg GD, Sklar P, State MW, Sutcliffe JS, Walsh CA, Scherer SW, Zwick ME, Barett JC, Cutler DJ, Roeder K, Devlin B, Daly MJ, Buxbaum JD: Synaptic, transcriptional and chromatin genes disrupted in autism. Nature 515:209–215, 2014 25363760 10.1038/nature13772 PMC4402723

[B5] Le Couteur A, Rutter M, Lord C, Rios P, Robertson S, Holdgrafer M, McLennan J: Autism diagnostic interview: A standardized investigator-based instrument. J Autism Dev Disord 19:363–387, 1989 2793783 10.1007/BF02212936

[B6] Lord C, Rutter M, Goode S, Heemsbergen J, Jordan H, Mawhood L, Schopler E: Autism diagnostic observation schedule: A standardized observation of communicative and social behavior. J Autism Dev Disord 19:185–212, 1989 2745388 10.1007/BF02211841

[B7] Stivaros S, Garg S, Tziraki M, Cai Y, Thomas O, Mellor J, Morris AA, Jim C, Szumanska-Ryt K, Parkes LM, Haroon HA, Montaldi D, Webb N, Keane J, Castellanos FX, Silva AJ, Huson S, Williams S, Gareth Evans D, Emsley R, Green J, Campbell S, Ellicott R, Harrison E, Kapasi A, Mercatali G, Moon R, Tobin H, Velandy S, Wagstaffe R, Burkitt-Wright E, Vassallo G, West S, Eelloo J, Hupton E, Patel S, Howard E, Tricker K, Lewis L, Dobbie A, Drimer R, Sharif SM, Bassi Z, Acharya J, Lam W, Harrower N, Quarrell O, Bradbury A, Splitt M, Musson S, Jones R, Bethell H, Prem C, Horridge K, Steiger C, Consortium S: Randomised controlled trial of simvastatin treatment for autism in young children with neurofibromatosis type 1 (SANTA). Mol Autism 9:12, 2018 29484149 10.1186/s13229-018-0190-z PMC5824534

